# Supplementary material for: Biomarkers of Micronutrients in Regular Follow-Up for Tyrosinemia Type 1 and Phenylketonuria Patients
Source: Nutrients. 2019 Aug 27;11(9):2011. doi: 10.3390/nu11092011 (PMC6769775; doi:10.3390/nu11092011)
Supplement: Supplementary file 1 [file nutrients-11-02011-s001.zip › nutrients-564228-SI.pdf]

Supplementary material S1. Micronutrients and minerals in the different amino acid mixtures used in our patient group.

**PKU**											
**Supplement**	**Per Unit**	**Sodium (mg)**	**Potassium (mg)**	**Chloride (mg)**	**Calcium (mg)**	**Phosphorus (mg)**	**Magnesium (mg)**	**Iron (mg)**	**Zinc (mg)**	**Copper (mg)**	**Manganese (mg)**
PKU anamix infant	100 gram powder	191	501	355	410	300	58	8.1	5.7	0.43	0.43
PKU squeezie	100 ml	107	264	164	306	233	47	4	3.1	0.22	0.48
PKU 2 shake	100 gram powder	<30	560	<30	670	395	140	7.9	7.6	0.6	1.1
PKU lophlex 20	Sachet (27.8 g)	<5.6	<2.8	<1.4	356	276	107	5.3	3.9	0.53	0.53
PKU lophlex 10 LQ	62.5 ml	<15	50	<15	178	138	53.5	2.6	1.9	0.26	0.26
PKU lophlex 20 LQ	125 ml	<25	100	<25	356	276	107	5.3	3.9	0.53	0.53
PKU 2 mix	100 gram powder	256	540	405	1035	620	142	12.2	8.9	0.96	1.62
PKU cooler 10	100 ml	60	140	80	230	205	63	4.2	3.2	0.42	0.48
PKU cooler 15	100 ml	60	140	80	230	205	63	4.2	3.2	0.42	0.48
PKU cooler 20	100 ml	60	140	80	230	205	63	4.2	3.2	0.42	0.48
PKU air 10	100 ml	60	140	80	230	210	63	4.2	3.2	0.42	0.29
PKU air 15	100 ml	60	140	80	230	210	63	4.2	3.2	0.42	0.29
PKU air 20	100 ml	60	140	80	230	210	63	4.2	3.2	0.42	0.29
PKU 3 advanta	100 gram powder	<20	1760	<20	1410	704	511	21	18	1.7	3.5
PKU 2 fruta	100 ml	<40	280	<20	336	198	70	4	3.8	0.29	0.57
PKU express 20	100 gram powder	508	940	728	1196	1068	376	21.6	21.6	2.2	3.2
Phlexy 10 tablets	10 tablets	<5	<5	178	<5		13.2				
Phlexy 10 drink	20 gram	<1									
Xphe smart A	100 gram powder	0			1090	733	312	21	11	1.8	5
Xphe mini's	100 gram		931		1651	652	401	19	9	1.6	4
Lophlex sensation	Package (109 g)	<20	98.1	<20	356	276	107	5.3	3.9	0.53	0.53
PKU 3 shake	Sachet (50 g)	<15	583	<15	423	223	179	7.03	5.29	0.59	1.12
**TYR**											
Tyr anamix Infant	100 gram	191	501	355	410	300	58	8.1	5.7	0.43	0.43
Tyr cooler 10	100 ml	60	140	80	230	205	63	4.2	3.2	0.42	0.29
Tyr cooler 15	100 ml	60	140	80	230	205	63	4.2	3.2	0.42	0.29
Tyr cooler 20	100 ml	60	140	80	230	205	63	4.2	3.2	0.42	0.29
Tyr 2 prima	100 gram powder	541	1200	902	2299	1380	315	27	19.8	2.1	3.6
Tyr gel	100 gram	379	938	583	1083	825	167	14	11	0.8	1.7

PKU										
**Supplement**	**Per Unit**	**Fluoride (mg)**	**Molybdenum (µg)**	**Selenium (µg)**	**Chromium (µg)**	**Iodine (µg)**	**Vitamin A (µg)**	**Vitamin D (µg)**	**Vitamin E (mg)**	**Vitamin K (µg)**
PKU anamix infant	100 gram powder		12	15.5	13.8	83	392	8.7	4.6	37.2
PKU squeezie	100 ml			10		39	169	4.1	2.6	11.5
PKU 2 shake	100 gram powder		35	24	17	92	430	4.3	5.8	20.6
PKU lophlex 20	Sachet (27.8 g)		25	26.7	10.6	58.4	285	3.6	3.2	24.9
PKU lophlex 10 LQ	62.5 ml		12.5	13.4	5.3	29.2	143	4	1.6	12.4
PKU lophlex 20 LQ	125 ml		25	26.8	10.6	58.4	285	8	3.2	24.9
PKU 2 mix	100 gram powder	0.7	43	24.3	40.5	146	810	18.9	10.8	25.2
PKU cooler 10	100 ml		13	15	8	49	150	5.8	3	14
PKU cooler 15	100 ml		13	15	8	49	150	5.8	3	14
PKU cooler 20	100 ml		13	15	8	49	150	5.8	3	14
PKU air 10	100 ml		13	15	8	49	150	5.8	3	14
PKU air 15	100 ml		13	15	8	49	150	5.8	3	14
PKU air 20	100 ml		13	15	8	49	150	5.8	3	14
PKU 3 advanta	100 gram powder	1.1	99	77	99	261	1130	28	16	77
PKU 2 fruta	100 ml		18	12	8.8	46	215	2.2	2.9	10.3
PKU express 20	100 gram powder		144	88	88	252	832	13.2	15.6	100
Phlexy 10 tablets	10 tablets									
Phlexy 10 drink	20 gram									
Xphe smart A	100 gram powder		105	54	70	246	1052	8	18	77
Xphe mini's	100 gram		93	48	62	217	931	7	16	68
Lophlex sensation	Package (109 g)		25	26.8	10.6	58.4	285	8	3.2	24.9
PKU 3 shake	Sachet (50 g)	0.33	29.7	23.2	38.5	78.4	340	8.4	4.96	12.1
**TYR**										
Tyr anamix Infant	100 gram		12	15.5	13.8	83	392	8.7	4.6	37.2
Tyr cooler 10	100 ml		13	15	8	49	150	5.8	3	14
Tyr cooler 15	100 ml		13	15	8	49	150	5.8	3	14
Tyr cooler 20	100 ml		13	15	8	49	150	5.8	3	14
Tyr 2 prima	100 gram powder	1.5	94.5	54	90	323	1800	42	19.2	51.1
Tyr gel	100 gram		50	35	71	138	600	14.6	9	41

Supplement	Per Unit	Vitamin B1 (mg)	Vitamin B2 (mg)	Vitamin B3 (mg)	Vitamin B5 (mg)	Vitamin B6 (mg)	Folic acid (µg)	Vitamin B12 (µg)	Vitamin B8 (µg)	Vitamin C (mg)
**PKU**										
PKU anamix infant	100 gram powder	0.5	0.5	7.5	2.8	0.5	55	1.2	18.2	49
PKU squeezie	100 ml	0.28	0.34	4	1.4	0.3	59	0.56	7.1	17.7
PKU 2 shake	100 gram powder	0.74	0.86	5.3	3.3	0.96	1189	1.4	28	42
PKU lophlex 20	Sachet (27.8 g)	0.42	0.5	7.1	1.8	0.58	249	1.8	53.4	17.8
PKU lophlex 10 LQ	62.5 ml	0.21	0.25	3.6	0.88	0.29	60	0.88	26.7	8.9
PKU lophlex 20 LQ	125 ml	0.43	0.5	7.1	1.8	0.58	120	1.8	53.4	17.8
PKU 2 mix	100 gram powder	1.03	1.1	19.3	5.1	0.95	130	2	27	41
PKU cooler 10	100 ml	0.4	0.44	2	1.1	0.5	58	0.9	7.5	21
PKU cooler 15	100 ml	0.4	0.44	2	1.1	0.5	58	0.9	7.5	21
PKU cooler 20	100 ml	0.4	0.44	2	1.1	0.5	58	0.9	7.5	21
PKU air 10	100 ml	0.4	0.44	2	1.1	0.5	58	0.92	7.5	21
PKU air 15	100 ml	0.4	0.44	2	1.1	0.5	58	0.92	7.5	21
PKU air 20	100 ml	0.4	0.44	2	1.1	0.5	58	0.92	7.5	21
PKU 3 advanta	100 gram powder	1.75	2	22.4	8.4	2.1	231	4.2	70	119
PKU 2 fruta	100 ml	0.37	0.43	2.6	1.6	0.5	59	0.7	14	21
PKU express 20	100 gram powder	2	2.3	24.8	8	2.8	400	4.8	188	108
Phlexy 10 tablets	10 tablets									
Phlexy 10 drink	20 gram									
Xphe smart A	100 gram powder	1.8	2	28	11	2	632	6	175	91
Xphe mini's	100 gram	1.6	1.7	25	9	1.7	559	6	155	62
Lophlex sensation	Package (109 g)	0.43	0.5	7.1	1.8	0.58	120	1.8	53.4	17.8
PKU 3 shake	Sachet (50 g)	0.52	0.6	6.72	2.52	0.62	69.2	1.25	21	35.8
**TYR**										
Tyr anamix Infant	100 gram	0.5	0.5	8.2	2.8	0.5	55	1.2	18.2	49
Tyr cooler 10	100 ml	0.4	0.44	2	1.1	0.5	58	0.9	7.5	21
Tyr cooler 15	100 ml	0.4	0.44	2	1.1	0.5	58	0.9	7.5	21
Tyr cooler 20	100 ml	0.4	0.44	2	1.1	0.5	58	0.9	7.5	21
Tyr 2 prima	100 gram powder	2.3	2.4	22.8	11.4	2.1	230	4.5	60	90
Tyr gel	100 gram	1	1.2	14	5	1.1	208	2	25	63
